# Correlation between the antibiotic resistance and virulence determinants of vancomycin-resistant enterococci: paradoxical involvement of *van*A in phenotypic resistance to teicoplanin

**DOI:** 10.1186/s13099-025-00776-3

**Published:** 2025-12-14

**Authors:** Rana M. Amr, Amr S. Bishr, Khaled M. Aboshanab, Nadia A. Hassouna

**Affiliations:** 1 Department of Microbiology and Immunology, Faculty of Pharmacy, Ain Shams University, Cairo, 11566 Egypt; 2 Department of Microbiology and Immunology, Faculty of Pharmacy and Drug Technology, Egyptian Chinese University, Cairo, 11786 Egypt

**Keywords:** Vancomycin resistance, Enterococcus, Virulence genes, Resistance genes

## Abstract

**Background:**

Antimicrobial resistance, particularly in clinical *Enterococcus* isolates, poses a serious global health threat because of difficult-to-treat nosocomial infections. The emergence of vancomycin-resistant enterococci (VRE), mediated by VanA or VanB operons, has significantly limited treatment options. This study aimed at identifying antibiotic resistance and virulence genes in enterococci and exploring potential correlations between these genetic traits.

**Methods:**

A total of 100 suspected enterococci were gathered from two hospitals and identified through phenotypic methods and the VITEK 2 Compact system. The Kirby-Bauer disk diffusion and MIC by microbroth dilution methods were employed for antimicrobial susceptibility. The gelatinase production and biofilm were evaluated phenotypically, while the presence of vancomycin resistance (*van*A, *van*B) and virulence (*esp*, *gel*E, *hyl*) genes was confirmed by PCR and sequenced for genetic characterization.

**Results:**

Sixty-five *Enterococcus* isolates were characterized, with *E. faecium* (50.7%) and *E. faecalis* (41.5%) being the predominant species. Linezolid, teicoplanin, and chloramphenicol still retain good activity with 6.15%, 10.7%, and 29.2% resistance, respectively. About 40% of isolates were VRE, and all harbored the *van*A gene. Biofilm formation and gelatinase production were most prevalent in *E. faecium* and *E. faecalis*, indicating enhanced virulence. Sequencing confirmed the chromosomal location and identity of the resistance and virulence genes, supporting their accurate detection and distribution among different *Enterococcus* species. Statistical analysis revealed that both *esp* and *gel*E genes were significantly associated with biofilm formation and gelatinase activity; however, *esp* showed a positive correlation with *van*A and vancomycin resistance, while *gel*E demonstrated a negative correlation. Even though *van*A is typically linked to high levels of resistance to both teicoplanin and vancomycin, only seven out of the twenty-six isolates that were *van*A-positive showed phenotypic resistance to teicoplanin.

**Conclusion:**

*Enterococcus faecium* and *E. faecalis* were identified as predominant multidrug-resistant species carrying multiple virulence determinants, with *esp* and *gel*E strongly linked to biofilm formation and gelatinase activity. Linezolid, teicoplanin, and chloramphenicol remained the most effective agents. Our findings demonstrate the coexistence of resistance and virulence traits, along with unexpected genotype–phenotype variations, underscoring the need for integrated molecular and phenotypic approaches in surveillance and clinical management.

**Supplementary Information:**

The online version contains supplementary material available at 10.1186/s13099-025-00776-3.

## Background

In recent times, the rise of antimicrobial resistance among clinically relevant pathogens has emerged as a significant global health threat. Of particular concern is the development of resistance among pathogenic enterococci even to the most potent antibiotics, thereby severely limiting treatment options and posing a critical challenge to modern medicine. *Enterococcus faecium* and *Enterococcus faecalis* are clinically important species that combine multidrug resistance with virulence traits, posing a major challenge in healthcare settings [1]. Enterococci, classified as opportunistic pathogens, represent a significant global contributor to nosocomial infections. Among the two predominant species, *E. faecalis* accounts for about 80–90% of human enterococcal infections, while *E. faecium* is accountable for the majority of the remainder [2]. Clinical manifestations of enterococcal infections include bacteremia, endocarditis, and urinary tract infections [3].

Vancomycin-resistant enterococci (VRE) were first identified in 1986, nearly three decades after the clinical introduction of vancomycin. The emergence of resistance is believed to have been driven primarily by the use of oral vancomycin in hospital settings for the treatment of antibiotic-associated diarrhea. Resistance to vancomycin is mediated by one of two functionally analogous operons, VanA or VanB. These operons represent highly advanced genetic resistance elements, indicating that they likely originated in other bacterial species and were subsequently acquired by enterococci through horizontal gene transfer [4].

The presence of virulence-associated genes that promote immune evasion and improve survival against antimicrobial drugs is known to be linked to bacterial multidrug resistance (MDR) [5]. Enzymes like gelatinase and hyaluronidase, which are crucial for tissue invasion, colonization, and bacterial adhesion to host surfaces, are produced by these genes and help the pathogen create biofilms [6–8]. One important adhesion molecule that is essential to *Enterococcus* colonization and biofilm development is the extracellular surface protein (ESP). The pathogenicity of *Enterococcus* species has been linked to secreted virulence factors such gelatinase (GelE) and hyaluronidase (Hyl) [7]. Although the virulence mechanisms of VRE are not yet fully understood, several factors, such as enterococcal surface protein, aggregation substance, gelatinase, hyaluronidase, and the collagen-binding adhesin, have been implicated in facilitating tissue colonization [9, 10]. This study aimed to evaluate the current antimicrobial susceptibility pattern, investigate the antibiotic-resistant genes and virulence genetic determinants in clinical *Enterococcus* isolates, particularly VRE, as well as to explore the potential relationship between these genetic determinants.

## Methods

### Collection of *enterococcus* isolates

A total of 100 presumptive clinical enterococci were obtained from urine samples of patients undergoing routine examinations et al.-Demerdash and Al Kasr Al-Einy hospitals over the period from December 2021 to August 2023. For isolation, the isolates were initially propagated in brain heart infusion (BHI) broth (Greater Manchester, UK) followed by streaking onto BHI agar plates. The resulting colonies appeared as small, white growths on the surface of the agar [6].

### Characterization of collected isolates:

Microscopic examination of the isolates was performed using Gram staining. The *Enterococcus* colonies were observed as Gram-positive cocci and were confirmed by catalase negativity and the ability to grow in 6.5% NaCl [11]. In the bile-esculin azide test, *Enterococcus* species produced pinpoint brownish colonies surrounded by a characteristic black precipitate [12]. The VITEK 2 Compact system (bioMérieux, France) was used to confirm identification following the manufacturers protocol. The system calibration was verified using *E. coli* ATCC 25922.

### Antibiotic susceptibility test

The Kirby-Bauer disk diffusion method on Mueller–Hinton agar (HiMedia, India) was used to test the obtained *Enterococcus* isolates for antimicrobial susceptibility. Vancomycin (30 μg), teicoplanin (30 μg), erythromycin (15 μg), ampicillin/sulbactam (10/10 μg), linezolid (30 μg), ciprofloxacin (5 μg), chloramphenicol (30 μg), and doxycycline (30 μg) (Oxoid, UK) were among the antibiotics tested against a bacterial suspension equal to 0.5 McFarland turbidity standard. The susceptibility to vancomycin was validated by determining the minimum inhibitory concentration (MIC) using the microbroth dilution method. Quality control was ensured using *S. aureus* ATCC® 29,213 and *E. faecalis* ATCC® 29,212, according to the CLSI guidelines [13]. Additionally, isolates were inoculated onto bile esculin azide agar supplemented with 6 μg/ml vancomycin (Analytical grade, Julphar, Emirates) and incubated aerobically at 37 °C for 24 h. Colonies exhibiting pinpoint brownish morphology with surrounding black halos were identified as vancomycin-resistant *Enterococcus* (VRE) strains [14, 15].

## Phenotypic detection of enterococcal virulence determinants

### Gelatinase activity

To assess gelatinase production, the pure colonies of *Enterococcus* spp. were inoculated into 5 mL of nutrient broth supplemented with 3% gelatin (El Gomhouria Co., Egypt). The cultures were incubated at 37 °C for 24 h. Following incubation, the tubes were transferred to refrigeration at 4 °C for 30 min. Gelatin hydrolysis, indicative of gelatinase activity, was confirmed by the presence of liquefaction in the medium. In contrast, the absence of enzymatic activity was indicated by a solidified medium [16].

### Assessment of biofilm formation in enterococcus isolates

Biofilm production by *Enterococcus* isolates was evaluated using a modified standard protocol [16, 17]. Briefly, pure colonies were inoculated into 10 mL of trypticase soy broth (TSB) (HiMedia, India) containing 1% glucose (El Gomhouria Co., Egypt) and incubated overnight at 37 °C using *S. aureus* ATCC®29,213 as for quality control. Following incubation, 20 μL of the culture was transferred into wells of a 96-well microtiter plate containing 180 μL of fresh TSB + 1% glucose, adjusted to a final concentration of 10⁸ CFU/mL (measured at 600 nm). The plate was incubated at 37 °C for 24 h. To get rid of non-adherent cells, wells were gently washed twice with phosphate-buffered saline (PBS) after incubation. The plate was then heat-fixed at 55 °C for 60 min. To stain the biofilm, 150 μL of 1% crystal violet solution (El Gomhouria Co., Egypt) was added to each well and left for 15 min. Excess stain was removed, and the wells were rinsed twice with sterile distilled water. To solubilize the bound dye, 150 μL of 95% ethanol was added and left for 30 min. An ELISA plate reader was used to quantify each wells optical density (OD) at 490 nm. Three standard deviations above the mean OD of the negative control was the OD cut-off value (ODc). The ability to create biofilms was categorized as follows: Strong: ODs > 4 × ODc; Moderate: 2 × ODc < ODs ≤ 4 × ODc; Weak ODc < ODs ≤ 2 × ODc; and non-biofilm producers: ODs ≤ ODc as previously reported [3, 6].

### Determination of resistance genes with the polymerase chain reaction

Genomic DNA was extracted using the genomic DNA extraction kit (Qiagen, Hilden, Germany) and stored at –20 °C until further use for amplification [18]. Isolates with MIC of 2 μg/mL or more for vancomycin were subjected to polymerase chain reaction (PCR) (Perkin-Elmer/Applied Biosystems) analysis to detect the presence of vancomycin resistance genes (*vanA* and *vanB*, which code for D-alanin-D-lactate ligase A and B, respectively) using primers listed in Table S1 [11]. The PCR reactions were prepared in a total volume of 20 μL, comprising 2 μL of template DNA (100 nM), 1 μL of each primer (25 pmol), 10 μL of Master Mix (2X), and 6 μL of nuclease-free water. The PCR conditions were performed as previously reported [19].

### Determination of virulence genes esp, hyl, and gelE by PCR

The PCR was employed to detect three virulence-associated genes, including *esp*, *gel*E, and *hyl,* coding for extracellular surface protein, hyaluronidase, and gelatinase, respectively. A single PCR reaction was employed to detect *esp*, *gel*E, and *hyl*. Gene-specific primers (Table S1) were used [11, 20]. For PCR reaction, a total of 20 μL reaction mixture was prepared, containing 2 μL of template DNA (100 nM), 1 μL of each specific primer (25 pmol each), 10 μL of Master Mix (2X), and 6 μL of nuclease-free water. PCR amplification was carried out as previously described [20].

### Detection of amplified products

The PCR products were analyzed through agarose gel electrophoresis, where a 0.8–1.0% agarose gel was prepared in 1X TAE buffer. Ethidium bromide (Fisher Scientific, UK) was incorporated into the gel at a final concentration of 0.1 μg/mL before solidification. The gel was cast in a tray fitted with an appropriate comb and allowed to polymerize at room temperature for 30–45 min. Upon solidification, the comb was carefully removed, and the gel tray was transferred into the electrophoresis chamber. The chamber was then filled with 1X TAE buffer to submerge the gel by approximately 1 mm. Test samples (15 μL each) were carefully loaded into designated wells using a micropipette. DNA fragment sizes were determined by comparison with a molecular weight marker (GeneRuler 1 kb DNA ladder, Thermo Fisher Scientific, Waltham, Massachusetts, USA) as previously reported [18].

### DNA sequencing of the PCR products

PCR amplification products were purified using the GeneJET™ Purification Kit (Thermo Fisher Scientific, Waltham, Massachusetts, USA). The purified DNA was subsequently sent for bidirectional sequencing (forward and reverse primers) at Macrogen (Korea) using the ABI PRISM 3730xl DNA Analyzer. Raw sequence data were assembled into consensus contigs utilizing the Bioedit software (version 3; http://staden.sourceforge.net/) (accessed on 15 May 2025). Open reading frames (ORFs) within the assembled contigs were identified using FramePlot version 2.3.2 (http://www0.nih.go.jp/~jun/cgi-bin/frameplot.pl) (accessed on 15 May 2025). [21]. Each predicted ORF was analyzed through BLAST for sequence similarity, functionally annotated [22] and the resulting annotated sequences were submitted to the GenBank database, where accession numbers were assigned.

### Phenotypic relatedness of collected clinical enterococcus isolates

Using Euclidean distances as previously described, the heatmap analysis was conducted using Morpheus online software to evaluate the phenotypic relatedness of the clinical *Enterococcus* isolates (https://software.broadinstitute.org/morpheus/) (accessed on May 2025) [23]. The antibiotic susceptibility, gelatinase, biofilm production, virulence, and vancomycin resistance genes served as the basis for the heatmap analysis.

### Statistical analysis

Statistical analyses were conducted using the Pearson Chi-square test. A *p-value* of ≤ 0.05 was interpreted as statistically significant. All statistical analyses were performed using SPSS software.

## Results

### Isolation and characterization of enterococci

Of the total bacterial isolates collected, sixty-five were identified as *Enterococcus* species based on several phenotypic characteristics, including Gram-positive cocci morphology, catalase negativity, growth in 6.5% NaCl, and the formation of brown to black colonies on Bile Esculin Agar (BEA). Species-level identification was confirmed using the VITEK 2 system, where 33, 27, 3, and 2 isolates, with percentages of 50.7%, 41.5%, 4.6%, and 3%, were identified as *E. faecium*, *E. faecalis*, *E. avium,* and *E. durans,* respectively.

### Determination of antibiotic sensitivity profiles

**Fig. 1:**
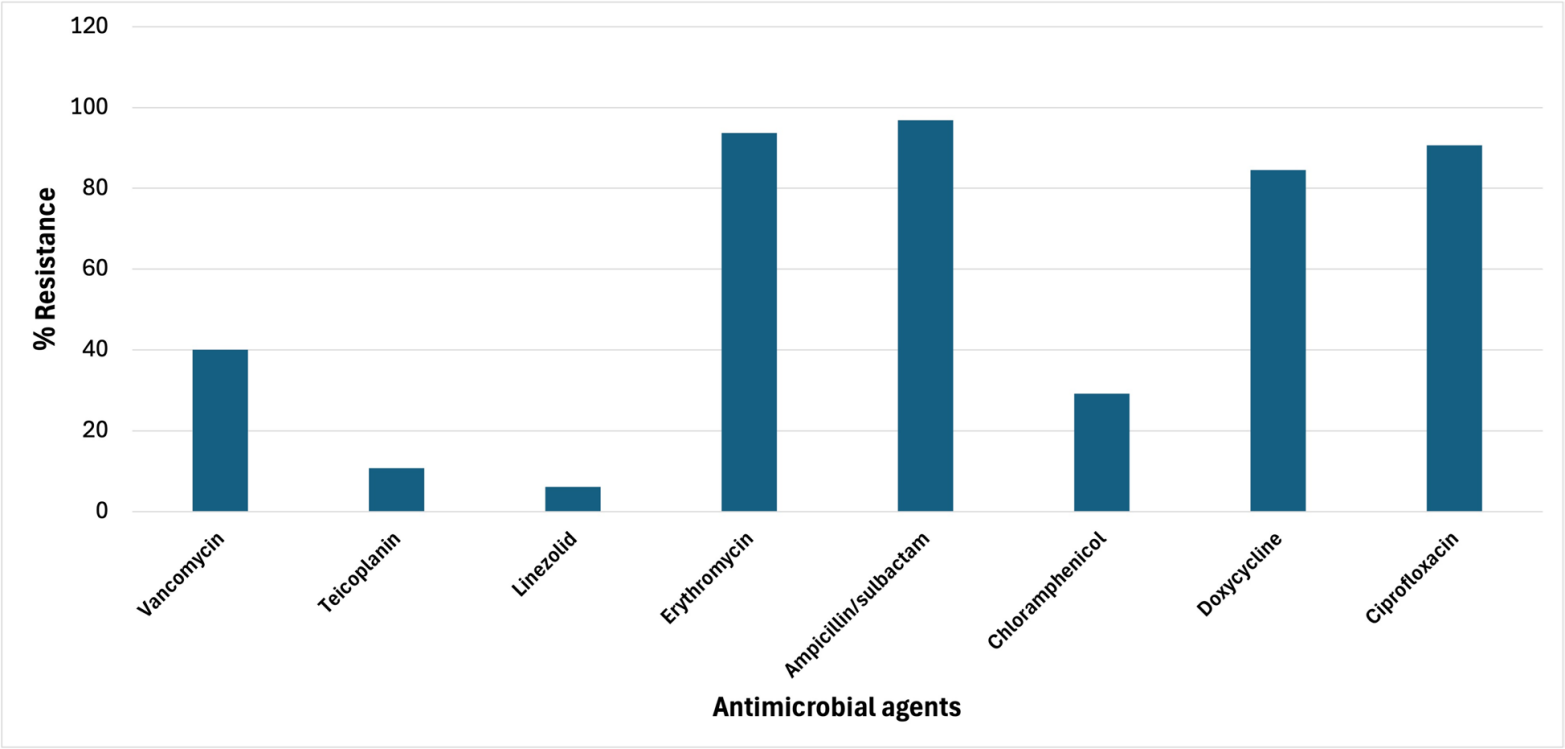
Percentage of antimicrobial resistance among the collected *Enterococcus* isolates (n = 65)

As depicted in Fig. 1, the highest rates of antimicrobial resistance were observed against ampicillin/sulbactam (96.9%; n = 63), followed by erythromycin (93.7%; n = 61), ciprofloxacin (90.6%; n = 59), and doxycycline (84.5%; n = 55) and vancomycin (40% resistance; n = 26). In contrast, the lowest resistance was recorded to linezolid (6.15% resistance; n = 4), teicoplanin (10.7% resistance; n = 7), and chloramphenicol (29.2% resistance; n = 19). Furthermore, 26 isolates (40%) were classified as VRE, while 39 isolates (60%) were identified as VSE, all VRE (40%, n = 26) exhibited a multidrug-resistant (MDR) phenotype. In contrast, within the VSE group (60%, n = 39), 31 isolates (79.5%) were MDR, while only 8 isolates (20.5%) remained non-MDR. This finding highlights that although MDR was universal among VRE, a substantial proportion of VSE also displayed resistance to multiple antimicrobial classes as shown in Table S2. Species-wise resistance analysis showed that *E. faecium* had the highest vancomycin resistance (60.6%), while *E. avium* (33.3%) and *E. faecalis* (18.5%) exhibited lower levels, and *E. durans* remained fully susceptible. Teicoplanin and linezolid resistance were rare, detected mainly in *E. faecium*. In contrast, resistance to erythromycin and ampicillin was widespread across all species, reaching 100% in *E. avium* and *E. durans*. High resistance to doxycycline and ciprofloxacin was observed in *E. faecium* and *E. faecalis*, and universally in *E. avium* and *E. durans*, whereas chloramphenicol resistance was less frequent (Table S3). The presence of VRE was further confirmed by PCR detection of *van*A or *van*B resistance genes in the respective isolates.

### Results of biofilm and gelatinase characteristics

**Fig. 2:**
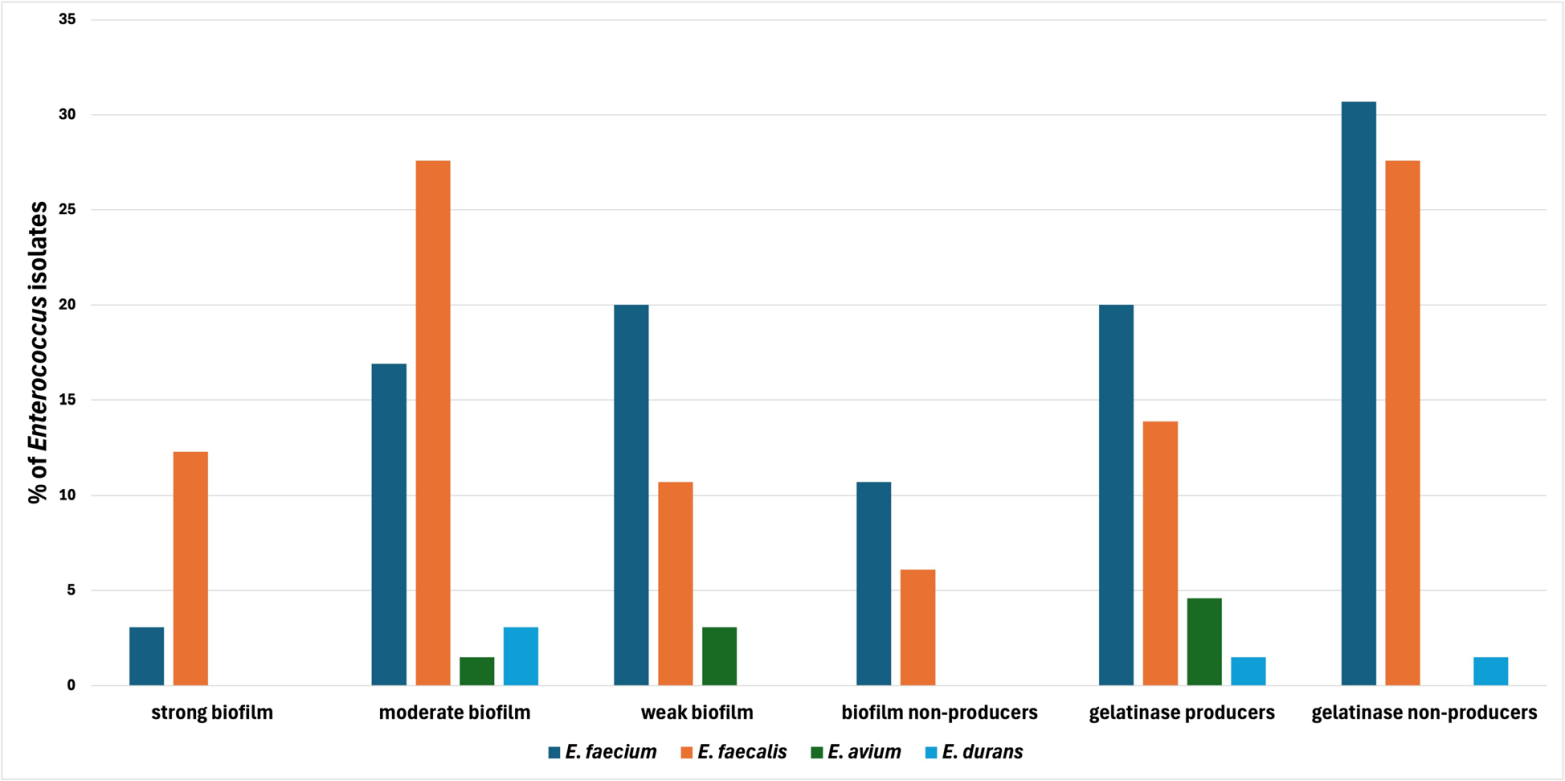
Percentage of biofilm and gelatinase producers among the collected clinical *Enterococcus* isolates (n = 65)

Of the 65 clinical *Enterococcus* isolates, 54 (83%) exhibited positive biofilm formation. The distribution among species (n = 54) was as follows: *E. faecium* accounted for 26 isolates (48.1%), *E. faecali*s for 23 isolates (42.6%), *E. avium* for 3 isolates (5.6%), and *E. durans* for 2 isolates (3.7%). The classification of the biofilm-forming ability of the collected isolates (strong, moderate, and weak) is displayed in Table 1. Regarding gelatinase production, 26 isolates (26/65; 40%) were positive. The highest prevalence was observed in *E. faecium* isolates showed the highest percentage (13/26; 50%), followed by *E. faecalis* (9/26; 34.6%), *E. avium* (3/26*;* 11.6%), and (1/26; 3.8%). These findings indicate that *E. faecium* and *E. faecalis* not only dominate in biofilm formation but also exhibit a higher frequency of gelatinase activity, suggesting their enhanced virulence potential compared to other *Enterococcus* species. The percentage and the pattern of biofilm and gelatinase producers among the collected clinical *Enterococcus* isolates (n = 65) are depicted in Fig. 2**.**

**Table 1:** Percentage of biofilm and gelatinase producers among the isolated clinical enterococci** (**n-65**)**

Isolate	Biofilm				Gelatinase	
	**Producers**			**Non-producers**	**Producers**	**Non-producers**
	**strong**	**moderate**	**weak**			
*E. faecium*	2	11	13	7	13	20
*E. faecalis*	8	8	7	4	9	18
*E. avium*	0	1	2	0	3	0
*E. durans*	0	2	0	0	1	1
Total	10	22	22	11	26	39
	54			11	65	
	65

### Resistance determinants analysis

**Fig. 3:**
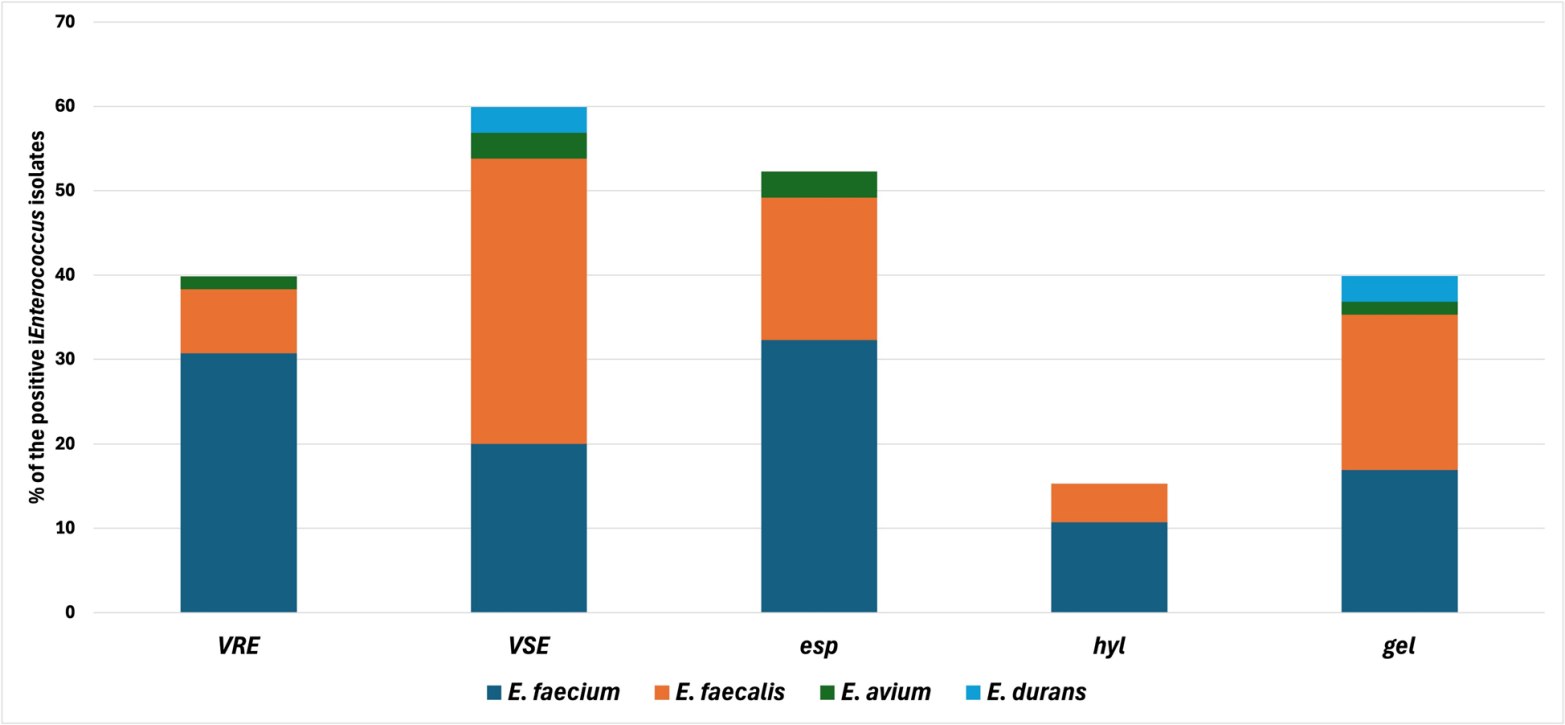
Percentage of the three virulence genes (*esp*, a virulence gene coded for extracellular surface protein; *gel*E, a virulence gene coded for gelatinase; *hyl,* a virulence gene coded for hyaluronidase) and the *Enterococcus* isolates (n = 65) exhibiting VRE and VSE phenotypes

Among the 65 *Enterococcus* isolates examined, 26 (40%) were classified as VRE, including 5 (7.7%) *E. faecalis*, 20 (30.8%) *E. faecium,* and 1 (1.5%) *E. avium*. On the other hand, 39 (60%) *Enterococcus* isolates were VSE (of which, 22 (33.8%) *E. faecalis*, 13 (20%) *E. faecium*, 2 (3.1%) *E. avium,* and 2 (3.1%)` *E. durans*. (Fig. 3). The VRE exhibited high-level resistance to vancomycin, with minimum inhibitory concentrations (MICs) ≥ 256 μg/mL. All VRE isolates harbored the *van*A gene, while the *van*B gene was not detected in any of them (Table S4).

### Detection of virulence genetic determinants in *enterococcus* spp

Among the *Enterococcus* isolates, the prevalence of virulence genes was as follows: *esp*, *gel*E, and *hyl* were detected in 34 (52.3%), 26 (40%), and 10 isolates, respectively (Fig. 3). Within *E. faecalis* isolates, the *gel*E gene was the most detected virulence determinant (12; 18.4%), followed by *esp* (11; 16.9%) and *hyl* (3; 4.6%). For *E. faecium*, results showed that the highest prevalence of *esp* (21; 32.3%), followed by *gel*E (11, 16.9%), and *hyl* (7; 10.7%). In *E. avium, esp* and *gel*E were detected in 2 and 1 isolates out of the three recovered isolates. For *E. durans,* the two obtained isolates were positive for the *gel*E gene, while both *esp* and *gel*E were not detected (Table S4).

### PCR sequencing analysis results

DNA sequence analysis was performed on PCR-amplified products targeting resistance and virulence genes from selected *Enterococcus* isolates. The sequencing results confirmed the presence of three virulence genes (*esp*, *hyl*, and *gelE*) as well as the *vanA* resistance gene, all located on the chromosomal DNA. The obtained amplicons corresponded to the expected product sizes: *vanA* (642 bp), *esp* (495 bp), *gelE* (279 bp), and *hyl* (251 bp). BLAST analysis demonstrated high sequence similarity with reference sequences deposited in GenBank, showing 100% identity with *vanA* (Accession No. PV632011), *gelE* (Accession No. PP757518), and *hyl* (Accession No. PP757517). The *esp* sequence also showed high similarity to its corresponding reference sequence. The final assembled sequences of vanA, esp, gelE, and hyl from selected E. faecium and E. faecalis isolates were deposited in the NCBI GenBank database and are available under the accession numbers PV696605, PV696608, PV696611, and PV696612, respectively.

### Phenotypic relatedness of collected clinical *enterococcus i*solates

**Fig. 4:**
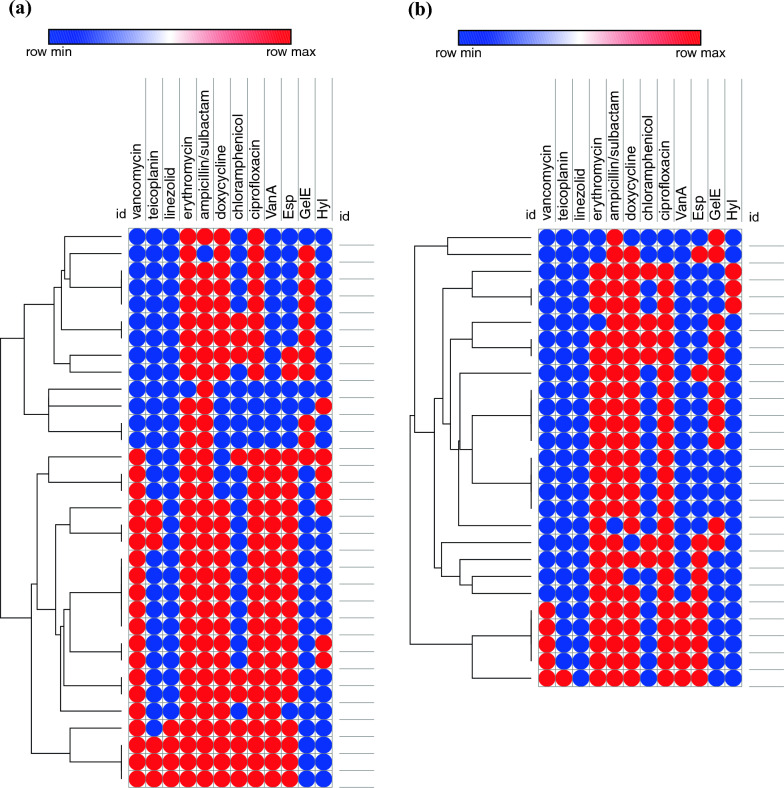
Heatmap analysis for phenotypic relatedness of: **(a)** *E. faecium* (n = 33) and **(b)** *E. faecalis* (n = 27) clinal isolates using Euclidean distances of Morpheus online software (https://software.broadinstitute.org/morpheus/)

As shown in Fig. 4a for *E. faecium* (n = 33) and Fig. 4b for *E. faecalis*, (n = 27), heatmap analysis revealed that the respective isolates were aligned in 19 and 16 groups, respectively, indicating their diversity and their non-clonal relatedness.

### Statistical correlation

Statistical analysis using SPSS demonstrated a significant positive association between the *esp* gene and both *vanA* carriage and vancomycin resistance (VRE) (p = 0.01), suggesting that *esp* may contribute to the enhanced pathogenic potential of resistant strains. In contrast, the *gelE* gene exhibited a significant negative correlation with *vanA* and VRE (p = 0.01), indicating that the presence of *gelE* was more common among vancomycin-susceptible isolates.This contrasting pattern indicates that the distribution of virulence genes is not uniform between resistant and susceptible *Enterococcus* populations and may be influenced by species-specific traits or regulatory mechanisms affecting gene carriage and expression.. In contrast, the presence of the *hyl* gene did not demonstrate a statistically significant correlation with the *van*A gene among VRE isolates (*p-value*, 0.16), suggesting that *hyl* may not play a comparable role in vancomycin resistance (Table 2). Additionally, there was a significant correlation between the *esp* and *gel*E genes and key virulence factors, including biofilm formation and gelatinase production. However, the *hyl* gene did not show a significant association with either biofilm formation or gelatinase activity (Table 3). These findings shall highlight the important role of ESP and GelE enzymes in both the virulence and antimicrobial resistance of *Enterococcus* species.

**Table 2:** Correlation between vancomycin resistance gene (*van*A) and three virulence genes (*esp, hyl* and *gel*E)

		Number of *esp* gene (%)		Total	P-value
		No	Yes		
Number of *van*A gene (%)	No	30 (46%)	9 (14%)	39 (60%)	0.01
	Yes	1 (1.5%)	25 (38.5%)	26 (40%)	
		Number of *gel*E gene (%)		Total	*P-value*
		No	Yes		
Number of *van*A gene (%)	No	14 (21.5%)	25 (38.5%)	39 (60%)	0.01
	Yes	25 (38.5%)	1 (1.5%)	26 (40%)	
		Number of *hyl* gene (%)		Total	*P-value*
		No	Yes		
Number of *van*A gene (%)	No	35 (54%)	4 (6%)	39 (60%)	0.16
	Yes	20 (31%)	6 (9%)	26 (40%)	

*esp*, a virulence gene coded for extracellular surface protein; *gel*E, a virulence gene coded for gelatinase; *hyl,* a virulence gene coded for hyaluronidase; *vanA*, vancomycin resistance gene coded for D-alanin-D-lactate ligase A

**Table 3:** Correlation between biofilm and gelatinase production and three virulence genes (*esp, hyl* and *gel*E)

		Number of *esp* gene (%)		Total	P-value
		No	Yes		
Number of biofilm-producer (%)	No	2 (3%)	9(14%)	11 (17%)	0.032
	Yes	29 (44.5%)	25 (38.5%)	54 (83%)	
		Number of *gelE* gene (%)		Total	*P-value*
		No	Yes		
Number of biofilm- producer (%)	No	11 (17%)	0 (0%)	11 (17%)	0.003
	Yes	28(43%)	26 (40%)	54 (83%)	
		Number of *hyl* gene (%)		Total	*P-value*
		No	Yes		
Number of biofilm-producer (%)	No	9 (14%)	2 (3%)	11 (17%)	0.778
	Yes	46 (70.7%)	8(12.3%)	54 (83%)	
		Number of *esp* gene (%)		Total	*P-value*
		No	Yes		
Number of gelatinase-producer (%)	No	14 (21.5%)	25 (38.5%)	39 (60%)	0.02
	Yes	17 (26.2%)	9 (13.8%)	26 (40%)	
		Number of *gelE* gene (%)		Total	*P-value*
		No	Yes		
Number of gelatinase-producer (%)	No	33 (50.8%)	6 (9.2%)	39 (60%)	0.001
	Yes	6 (9.3%)	20 (30.7%)	26 (40%)	
		Number of *hyl gene* (%)		Total	*P-value*
		No	Yes		
Number of gelatinase-producer (%)	No	32 (49.2%)	7 (10.8%)	39 (60%)	0.483
	Yes	23 (35.4%)	3 (4.6%)	26 (40%)	

*esp*, a virulence gene coded for extracellular surface protein; *gel*E, a virulence gene coded for gelatinase; *hyl,* a virulence gene coded for hyaluronidase

## Discussion

The purpose of this study was to explore the genes responsible for enterococcis virulence and antibiotic resistance and investigate any possible relationships between these genetic characteristics. This correlation would influence the treatment and infection control strategies for such life-threatening pathogens, particularly *E. faecium,* which is one of the ESKAPE pathogens of limited therapeutic options according to WHO reports [24]. Our findings contribute novel insights into the complex interplay between resistance and virulence mechanisms and highlight potential genotype–phenotype mismatches that challenge conventional diagnostic assumptions. There is a well-established link between bacterial multidrug resistance (MDR) and the presence of virulence-associated genes that facilitate immune evasion and enhance survival against antimicrobial agents [5]. These genes contribute to the pathogens ability to form biofilms and produce enzymes such as gelatinase and hyaluronidase, which play important roles in tissue invasion, colonization, and bacterial adherence to host surfaces [6]. The extracellular surface protein (ESP) is a key adhesion molecule that plays a crucial role in *Enterococcus* colonization and biofilm formation. Alongside ESP, secretory virulence mediators such as hyaluronidase (Hyl) and gelatinase (GelE) have been extensively implicated in potentiating the invasive and pathogenic capacities of these organisms [9]. Gelatinase, synergizing with ancillary peptide effectors, is postulated to exacerbate the virulence phenotype by promoting extracellular matrix degradation, facilitating immune evasion, and accelerating the systemic dissemination of enterococcal infections [25]. A preponderance of Enterococcus-associated pathologies spanning catheter-associated urinary tract infections, endocarditis, periodontitis, and device-mediated infections are intrinsically linked to the organism’s proclivity for biofilm genesis [26, 27]. These biofilms function as sophisticated biological fortresses, conferring protection against phagocytic clearance and impeding antibiotic penetration. Consequently, biofilm-producing enterococcal strains exhibit a heightened pathogenic trajectory and are frequently associated with recalcitrant and clinically challenging infections [28]. In recent years, there has been a notable rise in infections caused by vancomycin-resistant *E. faecium*. Surveillance data indicate that this trend is particularly evident in Central, Southern, and Eastern Europe, where 10% to 50% of *E. faecium* isolates have been reported [29].

This finding is consistent with our results, in which *E. faecium* accounted for approximately 78% of the VRE isolates. Based on the antibiotic susceptibility profiles, erythromycin, ampicillin, doxycycline, and ciprofloxacin exhibited the lowest levels of activity against the collected *Enterococcus* isolates. These findings are aligned with previous reports recording similar resistance patterns [6, 30]. Notably, linezolid remains one of the few effective therapeutic options available for treating VRE infections. However, concerningly, four isolates in this study were found to be resistant to linezolid, indicating the emergence of MDR strains and further limiting treatment options. One of the most notable findings in our study is the discrepancy between *van*A genotype and teicoplanin resistance. Although *van*A is classically associated with high-level resistance to both vancomycin and teicoplanin [31], only 7 of the 26 *vanA*-positive isolates exhibited phenotypic resistance to teicoplanin. This inconsistency could be attributed to regulatory mutations, reduced gene expression, or partial operon dysfunction mechanisms previously documented in *Enterococcus* spp.[32, 33]. Our data reinforce the importance of integrating phenotypic susceptibility testing with genotypic screening to avoid under or overestimating resistance patterns in clinical diagnostics. The results demonstrated that among the Enterococcus isolates, the *esp* gene was the most prevalent, followed by *gel*E*,* while *hyl* was the least frequently detected. This distribution pattern is aligned with the results reported by Fahmy et al. [34] According to our results, we deduce that the prevalence of *esp* gene was significantly higher in (VRE) isolates compared to VSE isolates, which is congruent with Biswas et al. [35] and Vankerckhoven et al. [20].In contrast, the *gel*E gene was significantly more frequent in VSE isolates (p = 0.01), a finding that aligns with previous reports noting its reduced prevalence or absence among certain VRE collections [1]. This discrepancy may be partly explained by species distribution, as VRE isolates are predominantly associated with *E. faecium*, whereas *gel*E is more commonly harbored by *E. faecalis*. Thus, the lower frequency of *gel*E in VRE likely reflects the species composition of resistant versus susceptible populations[2].

A substantial proportion of *E. faecium* isolates were found to harbor the *esp* gene, whereas *gelE* and *hyl* were detected at much lower frequencies in this species. In contrast, *E. faecalis* isolates showed a higher prevalence of the *gel*E gene compared to *esp* and *hyl*, indicating species-specific differences in virulence gene distribution [9, 36]. In our study, the hyaluronidase gene (*hyl*) was detected in 10 isolates (15.3%). Specifically, it was identified in 3 (11.1%) of *E. faecalis* isolates and 7 (21.2%) of *E. faecium* isolates, indicating a higher prevalence among *E. faecium*. These findings are consistent with some previous studies reporting low detection rates of *hyl* in *E. faecalis* [37]. However, they contrast with the results of Jovanovic et al. [38], who reported that *hyl* was not detected in *E. faecalis* isolates. Our findings, consistent with previous studies, support the significant role of the *esp* gene in promoting biofilm formation [39–41]. Additionally, the *gel*E gene appears to contribute significantly to both biofilm development and the pathogenicity of enterococci, and this aligns with Hashem et al. [3]. These findings contrast with those of Mobarez et al., who reported that the presence of *esp* and *gel*E genes was neither necessary nor sufficient for biofilm production in enterococci [42].

In this study, heatmap analysis demonstrated that the obtained clinical *Enterococcus* isolates were aligned in 19 and 16 groups, respectively, for *E. faecium* (n = 33) and *E. faecalis* (n = 27), demonstrating their diversity and non-clonal relatedness [43]. The phenotypic unrelatedness is a very crucial step that should be confirmed before studying the correlation between virulence and genetic determinants to ensure effective statistical analysis. Correlation analysis between the presence of virulence genes and their phenotypic expression revealed that gelatinase activity was detected in 40% (26/65) of the clinical *Enterococcus* isolates. Interestingly, the *gel*E gene was also present in 40% (26/65) of isolates; however, only 77% (20/26) showed concordance between gene presence and phenotypic expression. The remaining 23% (6/26) of *gel*E-positive isolates did not exhibit gelatinase activity, suggesting that *gel*E may be transcriptionally silent or under regulatory suppression in these strains. This partial concordance highlights the complexity of gelatinase regulation and cautions against relying solely on genotypic detection to predict virulence. Moreover, an unexpected observation emerged among the *gel*E-negative but gelatinase-positive isolates. Specifically, 5 of the 6 discordant isolates were found to carry the *esp* gene, and statistical analysis confirmed a significant association between *esp* presence and gelatinase activity (*p value* = 0.02). This finding raises the novel possibility that *esp*, beyond its well-established role in biofilm formation and adhesion, may contribute directly or indirectly to gelatinase expression in the absence of *gelE*. These results challenge the conventional understanding that gelatinase production in *Enterococcus* is solely dependent on *gelE* and suggest a more intricate regulatory network potentially involving alternative genes or compensatory mechanisms. This stands in contrast to earlier studies, such as Saffari et al. [25], which concluded that *gelE* was not essential for gelatinase activity but did not explore the compensatory role of *esp*. To our knowledge, this is the first report to propose a potential link between *esp* and gelatinase expression, warranting further mechanistic studies to elucidate this relationship. In addition to genotype–phenotype correlations, our study revealed an unexpected phenotypic diversity among genetically similar isolates, particularly in biofilm formation and enzyme activity, despite harboring identical virulence gene profiles. This observation suggests that virulence expression in *Enterococcus* is likely modulated by multifactorial regulatory pathways beyond the mere presence of target genes. Such epigenetic or environmental regulation has been hypothesized in prior studies [44], but our findings offer empirical support for this concept in clinical isolates. Furthermore, the clustering analysis demonstrated notable genetic heterogeneity among isolates with similar resistance and virulence patterns, reinforcing the notion that pathogenic behavior is not solely determined by genetic content, but also by strain-specific regulatory mechanisms. This adds a novel dimension to the current understanding of enterococcal pathogenesis, emphasizing the need to explore transcriptomic and proteomic profiles in future studies.

Recent studies from Egypt and beyond demonstrate that vancomycin‐resistant *Enterococcus* (VRE) is not confined to clinical settings but also circulates in food-producing animals and animal‐derived products, highlighting the risk of cross‐species transmission and the One Health dimensions of antimicrobial resistance. For instance, a recent study of retail raw cow’s milk in Egypt reported that 24% of samples yielded VRE isolates, many of which exhibited co‐resistance to teicoplanin and linezolid, and harbored virulence genes such as *esp* and *gelE*. [3]Similarly, another study investigating VRE in chicken, dairy, and human sources in Egypt found high prevalence of *vanA* and *vanB* genes among isolates from both animal and human origins, and noted that the virulence gene *gelE* was almost universally present. [4]These findings echo our present observation of high virulence gene carriage and substantial phenotypic resistance among *Enterococcus* isolates. Together, these results reinforce that surveillance of VRE must extend beyond hospitals to food chains and animal products. The presence of VRE in retail foods could serve as a reservoir that contributes to clinical infections, either through direct exposure or via the food supply, underlining the importance of integrated monitoring and stricter controls in antimicrobial use across human and animal domains.

## Conclusion

This study examined and highlighted the antibiotic resistance and virulence gene distribution among clinical *Enterococcus* isolates, identifying *E. faecium* and *E. faecalis* as predominant species with high levels of MDR phenotype. Linezolid, teicoplanin, and chloramphenicol were still effective with only 6.15%, 10.7%, and 29.2% resistance. A significant proportion (40%) of isolates were vancomycin-resistant, all harboring the *van*A gene (coding for vancomycin resistance gene coded for D-alanin-D-lactate ligase A), which was strongly associated with the virulence genes *esp* (coding for extracellular surface protein) and *gel*E (coding for gelatinase). The *esp* gene was most prevalent and significantly correlated with biofilm formation and gelatinase production, highlighting its role in pathogenicity. DNA sequencing confirmed the chromosomal presence of *van*A, *esp*, *gel*E, and *hyl* (coding for hyaluronidase) with notable species-specific variation in virulence gene distribution, highlighting their critical role in enterococcal pathogenicity. Our studys most noteworthy discovery is the difference between teicoplanin resistance and the *van*A genotype. Even though, *van*A is typically linked to high levels of resistance to both teicoplanin and vancomycin, only seven out of the twenty-six isolates that were *van*A-positive showed phenotypic resistance to teicoplanin. This discrepancy may be explained by regulatory mutations, decreased gene expression, or partial operon dysfunction, all of which have been linked to *Enterococcus* species in the past. Our findings highlight how crucial it is to combine genotypic screening and phenotypic susceptibility testing to prevent clinical diagnostics from under- or overestimating resistance patterns.

## Supplementary Information

Additional file 1.

## Data Availability

All data generated or analyzed during this study are included in this published article and supplementary file. The *van* A, *esp*, *hyl,* and *gel* E nucleotide sequences of some selected E. *faecium* and *E. faecalis* clinical isolates were submitted to the NCBI GenBank under this submission PV696605, PV696606, PV696607, PV696608, PV696609, PV696610, PV696611, and PV696612, respectively.

## Abbreviations

BEA: Bile Esculin Agar
BHI: Brain heart infusion
CLSI: Clinical Laboratory Standards Institute
ESP: Enterococcal Surface Protein
ESKAPE: Is an acronym that combines the names of these bacteria: Enterococcus faecium, Staphylococcus aureus, Klebsiella pneumoniae, Acinetobacter baumannii, Pseudomonas aeruginosa, and Enterobacter spp
GelE: Gelatinase
Hyl: Hyaluronidase
MDR: Multidrug resistance
MIC: Minimum inhibitory concentration
NCBI: National Center for Biotechnology Information
PCR: Polymerase chain reaction
TAE: Tris Acetate EDTA
vanA: Vancomycin resistance gene coded for D-alanin-D-lactate ligase A
vanB: Vancomycin resistance gene coded for D-alanin-D-lactate ligase B
VRE: Vancomycin-resistant enterococci
VSE: Vancomycin-susceptible enterococci
WHO: World Health Organization
