# Supplementary material for: Correlation between the antibiotic resistance and virulence determinants of vancomycin-resistant enterococci: paradoxical involvement of vanA in phenotypic resistance to teicoplanin
Source: Gut Pathog. 2025 Dec 14;17:110. doi: 10.1186/s13099-025-00776-3 (PMC12744406; doi:10.1186/s13099-025-00776-3)
Supplement: Supplementary file 1 — Additional file 1. [file 13099_2025_776_MOESM1_ESM.docx]

**Table S1.** Primer sequences, annealing temperatures (T_a_), and expected product sizes for the resistance and virulence genes used in this study**.**

| **Gene name** | **Oligonucleotide sequence (5’ to 3’)** | **PCR Product size** | **T_a_ (ºC)** | **Reference** |
| --- | --- | --- | --- | --- |
| *van*A-F | GGGAAAACGACAATTGC | 732 | 55 | [8] |
| *van*A-R | GTACAATGCGGCCGTTA |  |  |  |
| *van*B-F | ATGGGAAGCCGATAGTC | 635 |  |  |
| *van*B-R | GATTTCGTTCCTCGACC |  |  |  |
| esp F | AGATTTCATCTTTGATTCTTGG | 510 |  | [8] |
| esp R | AATTGATTCTTTAGCATCTGG |  |  |  |
| *hyl* F | ACAGAAGAGCTGCAGGAAATG | 276 |  | [17] |
| *hyl* R | GACTGACGTCCAAGTTTCCAA |  |  |  |
| *gelE* F | TATGACAATGCTTTTTGGGAT | 213 |  |  |
| *gelE* R | AGATGCACCCGAAATAATATA |  |  |  |

*esp*, a virulence gene coded for extracellular surface protein; *gel*E, a virulence gene coded for hyaluronidase; *hyl,* a virulence gene coded for gelatinase; Ta, annealing temperature**;** *vanA*, vancomycin resistance gene coded for D-Alanin-D-Lactate ligase A; *vanB*, vancomycin resistance gene coded for D-Alanin-D-Lactate ligase B.

**Table S2.** Antibiotic susceptibility of the recovered. *Enterococcus* isolates (n= 65)**.**

| **Antimicrobial agent** | **Susceptibility** | **No of isolates (%)** |
| --- | --- | --- |
| Vancomycin | R | 26(40%) |
|  | I | 0(0%) |
|  | S | 39(60%) |
| Teicoplanin | R | 6(9.2%) |
|  | I | 1(1.53%) |
|  | S | 58(89.2%) |
| Linezolid | R | 4(6.1%) |
|  | I | 0(0%) |
|  | S | 61(93.8%) |
| Erythromycin | R | 59(90.7%) |
|  | I | 2(3.07%) |
|  | S | 4(6.15%) |
| Ampicillin/sulbactam | R | 63(96.9%) |
|  | I | 0(0%) |
|  | S | 2(3.07%) |
| Chloramphenicol | R | 11(16.9%) |
|  | I | 8(12.3%) |
|  | S | 46(70.7%) |
| Doxycycline | R | 53(81.53%) |
|  | I | 2(3.07%) |
|  | S | 10(15.38%) |
| Ciprofloxacin | R | 57(87.6%) |
|  | I | 2(3.07%) |
|  | S | 6(9.2%) |

S, susceptible; I, intermediate; R, resistant

**Table S3.** Species-wise distribution of antimicrobial resistance among *Enterococcus* isolates

| **Species** | **VAN (%)** | **TEI (%)** | **LZD (%)** | **ERY (%)** | **SAM (%)** | **DOX (%)** | **CMP (%)** | **CIP (%)** |
| --- | --- | --- | --- | --- | --- | --- | --- | --- |
| ***E. avium*** | 33.33 | 0.00 | 0.00 | 100.00 | 100.00 | 100.00 | 33.33 | 100.00 |
| ***E. durans*** | 0.00 | 0.00 | 0.00 | 100.00 | 100.00 | 100.00 | 50.00 | 50.00 |
| ***E. faecalis*** | 18.52 | 3.70 | 0.00 | 85.19 | 96.30 | 85.19 | 18.52 | 88.89 |
| ***E. faecium*** | 60.61 | 15.15 | 12.12 | 93.94 | 96.97 | 75.76 | 12.12 | 87.88 |

**Table S4**. Distribution of resistance (*van*A, *van*B) and virulence-associated genes (*esp, gel, hyl*) among *Enterococcus* species

| **Isolate code** | **Isolate name** | **vancomycin resistant genes** | | **virulence-associated genes** | | | **Phenotypic tests** | |
| --- | --- | --- | --- | --- | --- | --- | --- | --- |
|  |  | ***vanA*** | ***van B*** | ***esp*** | ***gel*** | ***hyl*** | **gelatinase production** | **biofilm production** |
| E1 | *E. faecalis* | - | - | - | + | - | + | + |
| E2 |  | + | - | + | - | - | - | + |
| E5 |  | - | - | - | - | + | + | + |
| E6 |  | - | - | - | + | - | - | + |
| E11 |  | - | - | - | + | - | + | + |
| E14 |  | - | - | + | + | - | - | + |
| E15 |  | - | - | - | - | - | - | + |
| E16 |  | - | - | - | - | + | - | + |
| E21 |  | + | - | + | - | - | + | + |
| E24 |  | - | - | + | + | - | - | + |
| E26 |  | - | - | + | + | - | - | + |
| E28 |  | - | - | - | + | - | + | + |
| E31 |  | - | - | - | + | - | + | + |
| E33 |  | - | - | - | - | - | - | + |
| E40 |  | - | - | - | + | - | + | + |
| E41 |  | - | - | + | - | - | - | + |
| E42 |  | - | - | - | + | - | + | + |
| E43 |  | - | - | - | + | - | - | + |
| E46 |  | - | - | + | - | - | - | + |
| E47 |  | - | - | - | - | + | - | + |
| E48 |  | + | - | + | - | - | - | - |
| E49 |  | - | - | + | - | - | - | - |
| E51 |  | + | - | + | - | - | - |  |
| E53 |  | - | - | - | - | - | - | - |
| E54 |  | + | - | + | - | - | - | - |
| E55 |  | - | - | - | + | - | + | + |
| E60 |  | - | - | - | - | - | - | + |
| E3 | *E. faecium* | + | - | + | - | + | - | + |
| E4 |  | + | - | + | - | - | + | + |
| E7 |  | - | - | - | - | - | - | + |
| E8 |  | - | - | - | + | - | + | + |
| E9 |  | - | - | - | + | - | + | + |
| E10 |  | + | - | + | - | + | - | + |
| E12 |  | - | - | - | - | - | - | + |
| E13 |  | + | - | + | - | - | - | + |
| E17 |  | + | - | + | - | - | - | + |
| E18 |  | + | - | + | - | - | - | + |
| E20 |  | + | - | + | + | + | + | + |
| E22 |  | + | - | + | - | + | - | + |
| E23 |  | + | - | + | - | + | + | - |
| E25 |  | - | - | + | + | - | + | + |
| E27 |  | - | - | - | + | - | + | + |
| E30 |  | - | - | - | + | - | + | + |
| E32 |  | - | - | - | + | - | + | + |
| E34 |  | - | - | - | + | - | + | + |
| E36 |  | - | - | - | - | + | - | + |
| E37 |  | - | - | - | + | - | + | + |
| E44 |  | - | - | + | + | - | + | + |
| E45 |  | - | - | - | + | - | + | + |
| E50 |  | + | - | + | - | - | - | - |
| E52 |  | + | - | + | - | - | - | + |
| E56 |  | + | - | + | - | - | - | + |
| E57 |  | + | - | + | - | - | - | - |
| E58 |  | + | - | + | - | - | - | - |
| E59 |  | + | - | + | - | - | - | - |
| E61 |  | + | - | + | - | - | - | + |
| E62 |  | + | - | - | - | - | - | + |
| E63 |  | + | - | + | - | - | - | - |
| E64 |  | + | - | + | - | + | - | - |
| E65 |  | + | - | + | - | - | - | + |
| E19 | *E. avium* | + | - | + | - | - | + | + |
| E35 |  | - | - | - | + | - | + | + |
| E39 |  | - | - | + | - | - | + | + |
| E29 | *E. durans* | - | - | - | + | - | - | + |
| E38 |  | - | - | - | + | - | + | + |

(+) means positive PCR product or positive phenotypic test; (-) means negative PCR product or negative phenotypic test. *esp*, a virulence gene coded for extracellular surface protein; *gel*E, a virulence gene coded for gelatinase; *hyl,* a virulence gene coded for hyaluronidase; Ta, annealing temperature**;** *vanA*, vancomycin resistance gene coded for D-Alanin-D-Lactate ligase A.
